# C-955-08. Integrating Burn Disaster Training with AHA Algorithms to Improve Communication, Education, and Team Dynamics

**DOI:** 10.1093/jbcr/irag033.157

**Published:** 2026-04-06

**Authors:** Krystle Ferreira, Kristy Gauthier

**Affiliations:** University of Utah Health Burn Center, Salt Lake City, UT; University of Utah College of Nursing, Salt Lake City, UT

## Abstract

**Introduction:**

At our ABA-verified Burn Center, the need for ongoing disaster preparedness education became evident during the COVID-19 pandemic, when in-person classes transitioned to virtual formats. This shift, coupled with increased staff turnover, contributed to variability in team preparedness. Multiple incident reports identified medication safety concerns during critical events, highlighting gaps in communication, role clarity, and standardized use of resuscitation protocols. To address these challenges, we developed a quality improvement initiative integrating burn disaster simulation with American Heart Association (AHA) Advanced Cardiovascular Life Support (ACLS) and Pediatric Advanced Life Support (PALS) algorithms, while also embedding Advanced Burn Life Support (ABLS) themes to emphasize burn-specific priorities.

**Methods:**

A multidisciplinary simulation program was implemented to combine realistic burn disaster scenarios with AHA algorithm–driven care. ABLS themes were incorporated to ensure burn-specific priorities such as airway management, fluid resuscitation, medication safety, and transfer considerations were embedded within each scenario. Simulations were designed to prepare nurses, providers, and interprofessional staff to care for the unique needs of burn patients in disaster situations. Participants completed pre- and post-training surveys using a 5-point Likert scale to assess confidence in caring for adult and pediatric patients, communication effectiveness, and team role clarity. Structured debriefings reinforced objectives and identified system-level opportunities for improvement.

**Results:**

Survey data demonstrated improvements across all domains. Confidence in managing burn patients during disasters increased from 3.61 to 4.09 (+0.48). Confidence in caring for adult patients rose from 3.93 to 4.23 (+0.30), and pediatric patients from 3.52 to 4.06 (+0.54). Team communication improved from 4.04 to 4.31 (+0.27), and role clarity from 3.84 to 4.26 (+0.42). Qualitative feedback emphasized the benefit of integrating burn-specific priorities with standardized resuscitation algorithms to strengthen interprofessional collaboration and situational awareness.

**Conclusions:**

Integrating burn disaster training with ACLS, PALS, and ABLS principles improved provider confidence, communication, and role clarity. This blended approach enhances team readiness to deliver consistent, evidence-based care during high-acuity burn events and supports system-wide disaster preparedness.

**Applicability of Research to Practice:**

The findings also provide a foundation for future research examining the impact of integrated algorithm-based training on clinical outcomes, medication safety, and interprofessional team performance during real-world burn mass casualty incidents.

**Funding for the study:**

N/A.

